# Combining environment and task manipulation improves the development of individual creativity in futsal players

**DOI:** 10.3389/fspor.2025.1532810

**Published:** 2025-04-01

**Authors:** Behzad Mohammadi Orangi, Behrouz Ghorbanzadeh, Mansoureh Shahraki, Mozhgan Memarmoghaddam, Matthieu Lenoir

**Affiliations:** ^1^ Department of Sport Science, Faculty of Humanities, Damghan University, Damghan, Iran; ^2^ Department of Physical Education and Sport Sciences, Faculty of Education and Psychology, Azarbaijan Shahid Madani University, Tabriz, Iran; ^3^ Department of Sport Sciences, Faculty of Literature and Humanities, University of Zabol, Zabol, Iran; ^4^ Department of Sports Biomechanics and Motor Behavior, Faculty of Sports Sciences, University of Mazandaran, Babolsar, Mazandaran, Iran; ^5^ Department of Movement and Sports Sciences, Ghent University, Gent, Belgium

**Keywords:** variability, constraints, education, coaching, degrees of freedom

## Abstract

**Introduction:**

Literature on the effect of task and environment manipulation with the purpose of stimulating creative actions in futsal is promising yet limited. The aim of this study was to compare the effect of task and environmental manipulation on individual creativity development of futsal players.

**Methods:**

To conduct this study, 40 male players of the university futsal teams (M_age_ = 23.99, SD = 2.21) were randomly divided into 4 training groups: (1) playing on the futsal field (G1—control), (2) Playing on the half-field futsal (G2—task manipulation), (3) Playing on the soccer field, simulated with futsal (G3—environment manipulation), and (4) Playing on half of the simulated field (G4—combined). The groups played futsal in 15 sessions according to the specific conditions of each group (5 × 5). Before and after the intervention, individual creativity was assessed by means of video analysis.

**Results:**

Players in G4 exhibited more progress at the level of total number of actions, and number of adequate, creative (G4: 1,300% increase >G2: 450% >G3: 300% >G1: 300%), and original (G4: 450% increase >G2: 171% >G1: 225% >G3: 71%) actions after the intervention. However, participants in G2 improved more with respect to the degree of variability in behavior (G2: 58% increase >G4: 53% >G1: 44% >G3: 21%).

**Discussion:**

These results emphasize the importance of manipulation of constraints (changing the size) as a potential gateway to stimulate creativity in futsal players. Moreover, this study highlights the significant role of combined manipulation (G4), which outperformed other groups in most creativity-related metrics, contributing valuable insights into futsal training methodologies.

## Highlights

• Changing the size of the land in the new environment promotes individual creativity.

• New positions may lead to increased variability and degrees of freedom, which causes the emergence of creative actions in futsal.

• Professional futsal players get the most benefit by playing in a different environment and a smaller field in terms of creating total, adequate, variability, original and creative actions.

## 1 Introduction

Stereotypically in motor learning, the basic idea is that practice in the same conditions as the competition (for example, futsal players should practice on the standard futsal field) facilitates transfer to the competition context. However, According to the ecological dynamics perspective in team sports, variability in training is a key factor in athletic success (1). Such variability enhances perception-action coordination by exposing athletes to new situations, improving their responsiveness and creativity. Additionally, task and environmental constraints encourage exploration of diverse actions, increasing the likelihood of discovering creative movement solutions (2).

Creativity is defined as functional movement patterns that are new to the individual or group in answering the limitations of a particular situation during the game (3), and is important in many areas of sports performance, and especially in team sports (4–7). Because in team sports, individual creativity can contribute to the success of the team (1). Individual creativity in team sports can be defined by the unique and original actions that players perform (8, 9). These actions can be categorized as creative (done by only 5% of the team), original (attempted by only 5% of the team), adequate (successful actions), and inadequate (unsuccessful actions) (8, 9). The greater the degree of variability of actions in total action within a team, the higher the level of individual creativity can be said to be. However creativity results in new actions emerging in a person as a result of exploratory practice (2). Based on the presented definition of creativity and previous studies (8–10), it is generally understood that a player in team sports like football and futsal possesses individual creativity when they exhibit the following characteristics: (1) They perform more creative and original actions during a game, (2) They make more appropriate decisions (adequate action), (3) They make fewer inappropriate decisions (inadequate action), and (4) They demonstrate a higher degree of variability in their actions (with at least six out of ten actions being different). From this definition of creativity, the need for practice designs that are challenging, promote exploratory behavior, and stimulate variability follows (11).

Degree of variability in practice is operationalized by manipulating the environment and the task (12). Task manipulation in the training environment refers to manipulation of the rules of any sport (13). Environmental constraints are defined as all factors that are external to the organism (14). Manipulation of the task and the environment are believed to improve the development of creativity in players^1^ (15). The claim of improving creativity by manipulating constraints has been proven to be promising in several experimental studies, although the evidence is still limited. In this regard, Canton et al. (16), in 6 vs. 6 small-sided games (SSGs), manipulated the task in soccer by changing the location of the goal and the number of players. Tactical and exploratory behaviors that are indicative of team creativity were positively affected by such adaptations. Similar findings were reported by Caso and van der Kamp (10). In this study, creativity and variability were compared in 5 × 5, 6 × 6, 7 × 7, and 11 × 11 games. The results showed that in SSG with fewer players, more creative actions emerged in more variable patterns.

A review of the proposed studies shows that task manipulation (changing the size of the playing field and the number of players) has a positive effect on motor creativity [e.g., (17)]. However, a more in-depth examination of the types of such manipulations can provide a new perspective on practice and training for enhancing motor creativity. In this regard, Santos et al. (18), demonstrated that using balls from different sports, such as handball, soccer, and rugby, has a greater impact on soccer performance than using soccer balls of different sizes. In this study, players trained in small-sided games (SSG) with 4 and 6 players using soccer balls (group 1), handballs (group 2), rugby balls (group 3), and a mix of these balls (group 4). The results showed that training under varied conditions can lead to performance improvement.

In the study by Santos et al. (18), the primary focus was on ball manipulation and playing field size (task manipulation). However, the key question is whether environmental manipulation (e.g., changing the playing field, such as training futsal players on a soccer field instead of changing the ball) can also be effective in this regard. Despite the importance of environmental factors, most studies have focused on task constraints. One reason for this is that task manipulation is easier to control than environmental manipulation. For instance, changing the size of the playing field or the number of players are variables that can be easily adjusted within a fixed environment. In contrast, changing the playing environment often comes with logistical and practical challenges (such as the need for different facilities, variations in playing surface material). As a result, many studies, such as Caso and van der Kamp (10), have only examined the effects of field size and the number of players on tactical and technical adaptations without altering the playing environment. Moreover, methodological limitations represent another barrier to investigating environmental manipulations, as comparing performance across different environments requires controlling for multiple factors that could influence the results. This research gap highlights the need to explore how transitioning from one sport's playing environment to another may impact motor creativity.

To address this issue, our study aims to clarify the relative contribution of task and environmental constraints to the emergence of creative behavior in futsal. Specifically, it examines the impact of task manipulation (changing the size of the playing field) and environmental manipulation (training futsal players on a soccer field) on the development of creative behaviors over a 15-session training period. Therefore, it is expected that both types of manipulations will influence the development of players’ individual creativity, as expressed through the number of actions, their variability, and the originality and adequacy of movements.

## 2 Methods

### 2.1 Participants

Based on a power analysis using G-Power and considering a 2 (pre-post) ×4 (groups) ANOVA test, a total of 40 participants were required for this study. The sample size calculation was based on an alpha level of 0.05, statistical power of 0.80 (1-β), and a moderate-to-large effect size (*f* = 0.40). These parameters were chosen in accordance with previous research on futsal interventions [as; (8, 9, 19)]. Therefore, 40 male futsal players of the university team participated in this study. To recruit participants, an announcement containing the aim of the study and the criteria to participate was advertised through university channels. Apart from being the university's futsal team member, inclusion criteria were (a) having a good physical and mental health status, based upon the student's health record, (b) completing written informed consent, and (c) not being involved in intensive training or competition while participating in this study. Participants playing at a higher level (e.g., at league level) were excluded in order to create a homogeneous group of participants with respect to expertise level. Among the 53 players who declared their readiness to participate in the research, 6 players were excluded because of playing at a higher level, 3 players because of not providing valid proof of membership in the university team, and 4 players were excluded because they were involved in other training and/or competition that might have affected their performance in the study. 40 selected participants took part in all training sessions, pre-test and post-test. Participants agreed not to engage in any additional interventions or exercises while participating in the study interventions. It should be noted that this study was conducted at the University of Zabol and the participants were members of teams from universities in Sistan and Baluchestan province. Before starting the research process, the proposal of this work was approved in the relevant faculty and ethical consent was received from all participants.

### 2.2 Procedure

The focus of this study was on the sport of futsal, which is played in a 5 vs. 5 player format. Therefore, in this study, 4 groups of 10 players (2 teams) were reacted [see (8, 9) for a similar procedure]. The players were randomly assigned to one of the four experimental groups by the second author, who had no prior knowledge of the players. The initial segmentation was conducted by the second author after the first author assigned a random number (from 1 to 40) to each participant through simple random sampling. These assigned numbers were then sent to the second author, who used a Random Sequence Generator (https://www.random.org/sequences/) to randomly allocate each player into one of the four groups. It is important to note that only the goalkeepers (numbers 1–4) were predetermined and each of them was randomly placed into a group during this process.

Group 1 (G1) played on the regular futsal field, group 2 (G2) played on half of the same futsal field (dividing the width and length of the regular futsal field in two). Group 3 (G3) was evaluated while playing on the football field, simulated with futsal (playing on the football field according to the rules of futsal), and group 4 (G4) played on the simulated half of the group 3 field (see Table 1 for more information). The descriptive information of the players is given in Table 2. As is evident from a One-way ANOVA, the groups could be considered as homogeneous with respect to age, mass, and body height.

**Table 1:** An overview of the group's conditions of practice.

Groups	Atmosphere	Surface	The light	Temperature	Width^a^ length	Number of gates and its width ^a^ length	Number of players	Ball
G1	Inside the futsal gymnasium (closed environment)	Parquet flooring	Artificial	Artificial	25 × 42 m	2 2 × 3 m	5 × 5	63.5 cm and 430 g
G2	Inside the futsal gymnasium (closed environment)	Parquet flooring	Artificial	Artificial	12.5/21 m	2 2 × 3 m	5 × 5	63.5 cm and 430 g
G3	Grass field for soccer (open environment)	Grass flooring	Natural	Natural	25 × 42 m	2 2 × 3 m	5 × 5	63.5 cm and 430 g
G4	Grass field for soccer (open environment)	Grass flooring	Natural	Natural	12.5/21 m	2 2 × 3 m	5 × 5	63.5 cm and 430 g

^a^ In this table, “natural” or “artificial” light and temperature refer to the lighting and temperature conditions within the hall. These conditions are controlled by devices such as lamps or heating/cooling systems within the hall, and by the sun outside the hall. We had no control over these factors and simply observed them as they were for the purposes of the study.

**Table 2:** Demographic characteristics of participants and one-way ANOVA test results for demographic information.

Variable	All *N* = 40 Mean ± SD	Group 1 *N* = 10 Mean ± SD	Group 2 *N* = 10 Mean ± SD	Group 3 *N* = 10 Mean ± SD	Group 3 *N* = 10 Mean ± SD	F	***p***
Age (years)	23.99 ± 2.21	24.12 ± 2.11	23.96 ± 2.14	23.74 ± 2.66	24.14 ± 1.96	0.82	0.54
Mass (kg)	67.96 ± 3.79	69.72 ± 4.02	68.01 ± 3.08	67.01 ± 4.85	67.13 ± 3.22	0.66	0.69
Height (cm)	178.9 ± 4.28	179.1 ± 7.47	178.4 ± 4.11	180.7 ± 3.14	177.4 ± 2.4	0.91	0.78

After the participants were divided into 4 groups, the necessary arrangements were made and the participants showed up for the pre-test in a rented gym one day. On the day of the pre-test, the G1 took the test at 4:00 p.m., the G2 at 5:30 p.m., the G3 at 7:00 p.m., and the G4 at 8:30 p.m. The pre-test of this study was only the futsal game in two halves of 20 min. The intervention consisted of 15 game sessions according to the conditions of each group, which started 3 days after the pre-test. The groups appeared in the pre-rented hall on Mondays, Wednesdays and Fridays in the following order. The G1 at 7 p.m., the G2 at 9 p.m, the G3 at 1 p.m. and the G4 at 3 p.m. The groups only played in 15 training sessions and no specific instructions were given, apart from trying to compete according to the rules of futsal and trying their best to win each game (similar to pre-test, post-test and intervention sessions). These games were played according to the rules related to futsal. Therefore, the training intensity was almost the same for all groups. The 36-year-old referee, holding a second-degree refereeing certification from the country's football federation, officiated the games. All games were coordinated and supervised by one of the authors. Each session consisted of one hour of play with 10 min of rest between halves in a competition format. Also, before each game, the players (individually and as a team) warmed up, which lasted 15 min on average. After the intervention, the post-test was exactly the same as the pre-test and 3 days after the intervention.

### 2.3 Assessment of creativity

In order to evaluate the motor creativity of players in the futsal game environment, four digital cameras installed from an elevation of approximately 3 m were filmed. Two cameras were placed behind the goals and two cameras parallel to the midline line of the field. The cameras were 25 Hz with a resolution of 2,448 × 3,264 megapixels [see (8, 9)]. It is important to note that only the pre-test and post-test were analyzed, and the intervention sessions were not included in the analysis. Accordingly, the examined variables were the total number of actions, diverse actions, adequate actions, inadequate actions, original actions, and creative actions.

To check futsal actions, all actions that a player can do in futsal have already been listed based on similar studies [see (8, 9)]. In this regard, the skills of passing, dribbling, shooting and trapping were identified as categories, each of which was divided into separate category. In total, 14 actions for passing, 9 actions for dribbling, 7 actions for shooting and 12 actions for trapping were identified [see Appendix 1, (8, 9)]. However, it was possible to identify new actions from the video recordings. In this study, we considered the total number of actions (all the actions that the players do), diverse (degree of variability: that is, actions that are diverse and not similar, for example, passing with outside and inside of foot are two different actions that are called 2 “degree of variability” action), adequate, inadequate, original and creative actions, referring to the study of Mohammadi Orangi, et al., (8, 9). Actions that were successful were considered as adequate actions (the pass goes to a teammate). Inadequate actions were actions that were performed by the player but were not productive (the pass does not reach the teammate). Actions that only 5% of players could do in a game (almost one person) were called original actions, and if they were successful, they were considered creative actions (8–10).

Two futsal analysts with more than 10 years of experience in this field analyzed all the videos. Before starting the coding process, they underwent a 1-week training session, which included familiarization with evaluation criteria, reviewing sample video clips, and discussing methods for identifying creative actions. To ensure reliability, both analysts independently coded 20% of the data. In cases of disagreement, discussions were held to resolve differences, and if no consensus was reached, the decision of the first analyst, who had more experience, was considered. Additionally, inter-rater agreement was assessed using kappa analysis, which indicated a high level of reliability (Cohen's *κ* = 0.79, *p* < 0.001, 95% CI: 0.75–0.91).

### 2.4 Statistical analysis

Kolmogorov Smirnov test was used to evaluate the normality of the data (*p* > 0.05). To evaluate the effect of the intervention on the number of total actions, variable and effective actions analysis of the 2 (pre-post) ×4 (groups) ANOVA test with the LSD *post hoc* test was used. For creative and original actions, due to the rarity of the results, they were reported descriptively. All data was analyzed on SPSS version 24 software. An alpha of 0.05 was used for all statistical tests. For effects size, partial eta squared are reported, with 0.14, 0.06 and 0.01 referring to large, moderate and small effect size, respectively (8, 9).

## 3 Results

**Figure 1:**
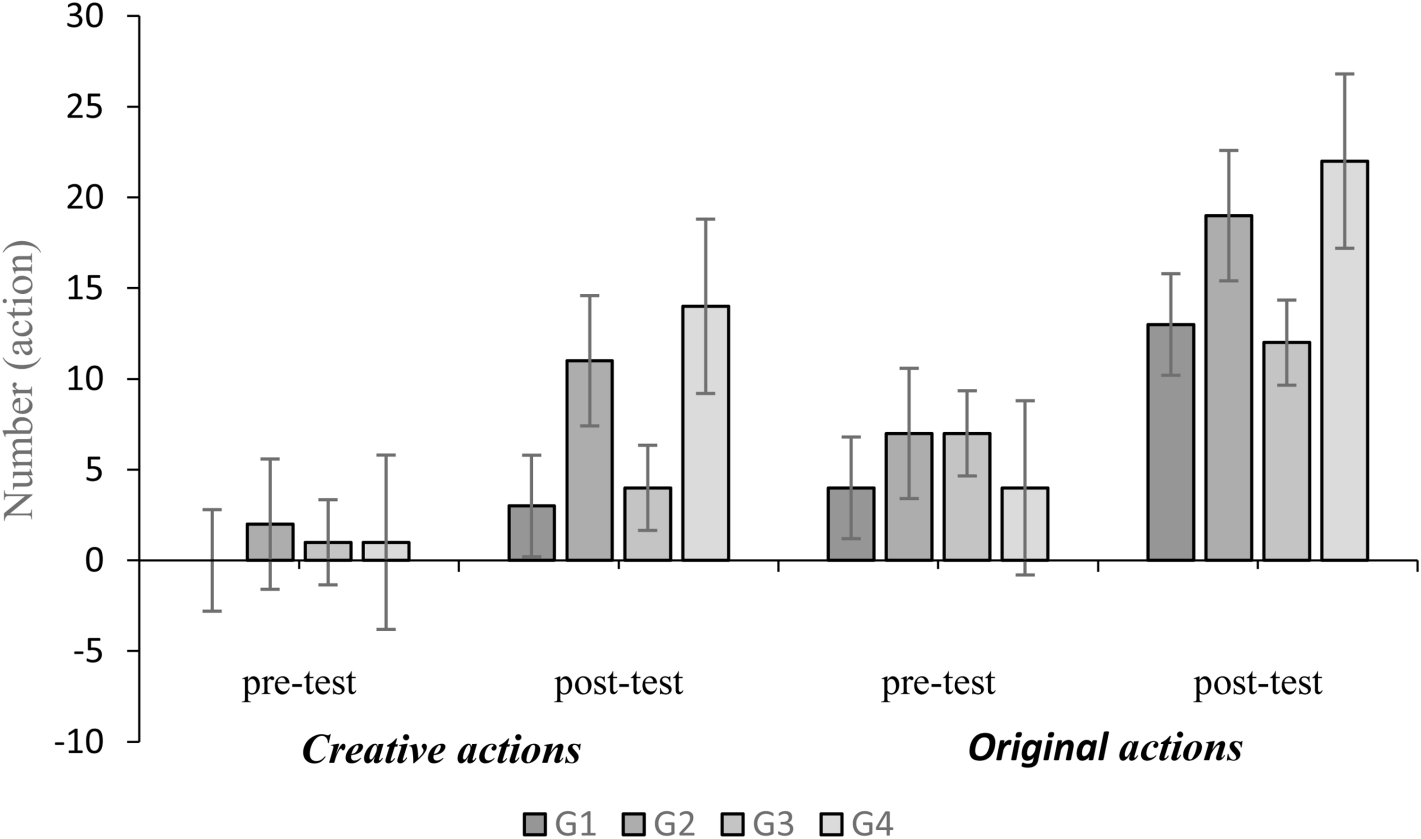
Creative and original actions in different groups in pre-test, and post-test.

The results of the Kolmogorov–Smirnov test showed that the data was normally distributed (*p* > 0.05). A total of 5,841 relevant actions were recorded across eight games, with 42.8% in the pre-test and 57.7% in the post-test. In the pre-test, 2,504 actions were identified, with 40% categorized as adequate and 15% as degree of variability. The post-test recorded 3,337 actions, where 45% were adequate and 16% had a degree of variability. Across all games, 88 actions were original, with 40% considered creative (4 in the pre-test, 32 in the post-test) (see Figure 1).

**Figure 2:**
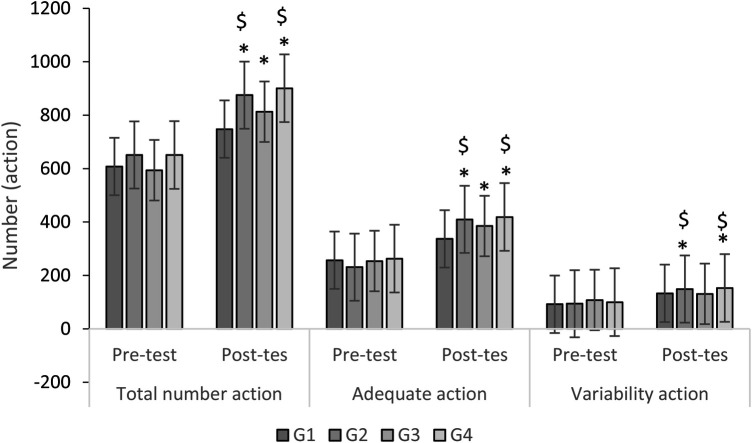
Total number, adequate, and degree of variability actions in different groups in pre-test, and post-test. *, significant difference with G1; $, significant difference with G3.

In the post-test, Group 4 (G4) had the highest share: 27% in total, adequate, and degree of variability actions (Figure 2), 33% in original actions, and 43% in creative actions (Figure 1). Table 3 presents the mean, standard deviation, and mean difference for these metrics. From pre-test to post-test, G4 led in total, adequate, original, and creative actions (G4 > G2 > G3 > G1), while G2 had the highest degree of variability (G2 > G4 > G1 > G3). See Appendix Table 1 for details.

**Table 3:** Descriptive for total number, adequate, and degree of variability.

Variable	Pre-intervention				Post-intervention				Mean difference			
	G1 (10)	G2 (10)	G3 (10)	G4 (10)	G1 (10)	G2 (10)	G3 (10)	G4 (10)	G1	G2	G3	G4
T	60.80 ± 5.53	65.10 ± 4.99	59.40 ± 4.83	65.10 ± 9.3	74.80 ± 3.32	87.5 ± 3.74	81.3 ± 4.66	90.1 ± 6.35	7.77	9.50	8.44	6.90
A	25.70 ± 4.11	23.10 ± 1.52	5.40 ± 3.06	26.3 ± 3.46	33.7 ± 2.66	41 ± 1.82	35.8 ± 2.3	41.9 ± 2.33	8.9	20.7	8.77	9.42
V	9.2 ± 2.74	9.4 ± 3.3	10.8 ± 2.89	9.85 ± 3.34	13.3 ± 1.33	14.9 ± 1.66	13.1 ± 1.66	15.3 ± 2.21	3.9	6.82	2.76	4.02

T, total number; A, adequate; V, degree of variability.

ANOVA (2 × 4) analysis revealed significant group-time interactions for total actions (F = 3.16, *P* = 0.036, *η*p² = 0.2), adequate actions (F = 14.21, *P* < 0.001, *η*p² = 0.54), and degree of variability (F = 2, *P* < 0.001, *η*p² = 0.14) (Table 4). No significant between-group differences were found in the pre-test for total (F = 2.09, *p* = 0.11), variable (F = 0.44, *p* = 0.72), and adequate actions (F = 1.93, *p* = 0.14). However, post-test results showed significant differences in total actions (F = 21.4, *p* < 0.001), degree of variability (F = 4.04, *p* = 0.014), and adequate actions (F = 29.9, *p* < 0.001).

**Table 4:** Overview of the outcomes of the analyses of variance for all dependent variables.

Variables	Statistics	Time	Group	Time × group	Effect sizes			
					D_G1_	D_G2_	D_G3_	D_G4_
Total number	F	243.44	15.99	3.16	2.45	3.0	2.67	2.19
	p	<0.001	<0.001	0.036				
	*η*p²	0.87	0.57	0.2				
Adequate	F	459.39	9.08	14.21	2.5	6.5	2.77	2.98
	p	<0.001	<0.001	<0.001				
	ηp²	0.92	0.43	0.54				
Degree of variability	F	68.69	0.72	2	1.16	2.15	0.87	1.27
	p	<0.001	<0.001	<0.001				
	ηp²	0.65	0.05	0.14				

*Post hoc* analysis showed significant group differences (*p* < 0.05) except between G2 and G4 for total, adequate, and degree of variability actions, and between G1 and G3 for degree of variability (*p* > 0.05) (see Table 5). G4 performed best, followed by G2, with G3 outperforming G1.

**Table 5:** Results of the *post hoc* test in comparison between the groups of variables in the post-test.

Condition	Total number (*p*)	Adequate (*p*)	Degree of variability (*p*)
G1 × G2	<0.001*	<0.001*	0.048*
G1 × G3	0.004*	0.049*	0.8
G1 × G4	<0.0011*	<0.001*	0.015*
G2 × G3	0.005*	<0.001*	0.027*
G2 × G4	0.221	0.338	0.612*
G3 × G4	<0.001*	<0.001*	0.008*

* Significance at the 0.05 level.

## 4 Discussion

The aim of this study was to compare the effect of task manipulation (changes in the size of the playing field) and environment manipulation (futsal vs. soccer physical environment) on the development of individual creativity of futsal players. In this study, we evaluated individual creativity based on the high number of individual actions in creative, original, appropriate actions, degree of variability in the total number of appropriate actions, and considered a person as having creativity that fits this definition [see (8–10)].

The results showed that practicing futsal on a soccer field half the size of the futsal field (G4—combined task and environment manipulation) was associated with the most progress in total, original, and creative actions from pre-test to post-test. Specifically, Group G4 showed a 1,300% increase in creativity compared to the pretest, while Group G2 (task manipulation only) demonstrated a 450% increase. In contrast, Group G3 (environment manipulation only) exhibited a 300% increase, indicating that environmental changes alone were not as effective in fostering creativity as the combined approach. Practicing in condition G2 was accompanied by a larger improvement in the number of adequate and degree of variability actions, with a 58% increase in variability compared to 53% in G4. In examining the difference between the groups, G4 was better than the other groups, followed by G2, G3, and G1 (control, without manipulation), respectively.

Conceptually, the lower effectiveness of environmental manipulation alone (G3) can be explained by the fact that while a new environment can introduce fresh stimuli and affordances, it does not inherently restrict or challenge players in a way that forces adaptive and creative problem-solving. Task manipulation, on the other hand, directly constrains the player's available space and options, compelling them to explore new movement solutions. When both environmental and task constraints are combined (as in G4), players experience both the novelty of a different setting and the functional challenge of limited space, which together create optimal conditions for creative development. In general, the results of this study indicated that changing the size of the playing field improves the development of individual creativity. Additionally, changing the playfield and moving to a newer environment can have a greater impact on the creative actions of futsal players when combined with task manipulation.

The change in the size of the playground and its effect on creativity have been examined in various studies, and almost all of them are in line with the results of the present study. In this regard, we can refer to Caso and van der Kamp (10), which showed that the small side game increases degree of variability and creativity in soccer players. Other studies (16, 20) also reported the positive effect of small side games on creativity. Regarding task manipulation, it can be said that sports such as soccer and futsal are unpredic, which require new actions from the player every moment (21). Undoubtedly, in such a sport, having a high freedom of action to perform variable actions will help the team. To achieve this goal, conditions must be created in the training and game environment that make the player perform degree of variability actions, and small side games have the ability to do this (10). Because in small side games one faces more action (i.e., has more chances to touch the ball). This opportunity further causes the person to be placed in a representative environment and this representativeness forces the person to perform creative action based on the person's perception of the environment (22).

With respect to the effect of environmental manipulation on the development of motor creativity, experimental studies are limited. The most interesting result of this study was the positive effect of the combination of environmental and task manipulation on the development of individual creativity. In this way, changing the environment of futsal players and taking them to the soccer field if it is accompanied by a small side game (i.e., G 4) improves the development of individual creativity. From the perspective of ecological dynamics (23), learning originates from the perception an individual has of the environment. Changing the environment affects this perception-action coupling, leading to different affordances that might support the learning of the individual. In this regard, the affordances present in the environment shape the actions of the learner/player to the new conditions so that the player is challenged to perform a movement appropriate to the environment (24). In our study, it was also confirmed that the players who played in the simulated environment were more creative than the players who trained in the real environment (G4 compared to G2 and G3 compared to G1). These results align with the Santos et al. (18), study which showed that using different sports balls, new and different conditions from the main conditions of a specific match/sport can improve performance. Therefore, it seems that matching the conditions of the competition and the game/practice is not always helpful as is sometimes advocated in traditional approaches to motor learning, because sports and especially team sports do not develop in a predictable manner, with the actions of the individual as well as the team are dependent on the actions of the other players on the field (25). Apart from the explanations above, changing task and environment might be beneficial in other ways too. New environments are always attractive to the learner (26, 27) and probably increase their motivation to continue the activity, and more efforts to learn. Also, in the sport of soccer, the grass surface might be considered a safer environment with respect to specific actions, in which the player is likely to do more unique moves (such as bicycle kick) without fear of falling or getting hurt, which leads to the emergence of creative actions in a person.

### 4.1 Strengths and weaknesses of this study

Based on the author’ information, this study was the first study that investigated the effect of task manipulation on the development of creativity in an environment different from the target sport. In this regard, combining task and environment manipulation and the obtained results might open new gateways to constraint manipulation for researchers and coaches. In this study, G-power was used to estimate the sample size based on previous studies (19). But the main limitation of the present study could be its small sample size. Undoubtedly, a small sample size may lead to false-positive results and inflated effect sizes (28). But every study with every limitation is needed to make an empirical estimate and to expand the new research vision in the future (29). Also, the number of samples in this study is proportional to the chosen sport (19). In this way, because in futsal there are 5 people in each team, we also tried to avoid other disturbing factors (equal opportunity to play for everyone) and we tried to divide each group into two halves of 5 people. Another limitation was that this study only focused on a limited number of constraints. Future studies could combine the current manipulations with changing the number of players. In addition, the current methodology did not allow us to evaluate the impact on tactical behavior and team creativity, which should be addressed in future studies. Finally, comparing futsal with other team sports in future studies could expand the results of this study. However, one should be cautious when generalizing these results due to the small sample size. Individual creativity is always linked to team creativity and should be evaluated together in future studies. Overall, this study will serve as a good starting point for further research.

## 5 Conclusion

In general, based on the results of this study, it can be concluded that changing the players’ environment can expose them to new conditions with new demands, requiring them to adapt accordingly. This process increases their degrees of freedom, encouraging them to perform creative and novel actions. Additionally, reducing the size of the playing field can lead players to engage in more creative movements due to spatial constraints, resulting in an increased number of actions. For futsal coaches, the findings of this study suggest that incorporating environmental manipulation in training sessions can be an effective strategy for enhancing players’ creativity. For instance, coaches can design drills that require players to train on fields of varying sizes or under different environmental conditions, such as playing on grass instead of indoors or practicing in smaller spaces to enhance tactical diversity and problem-solving in limited space. Furthermore, this study can serve as a foundation for further research. As a future research avenue, exploring the interaction between environmental modifications and other factors, such as the number of players, training duration, or the impact of specific training methods on creativity development, could provide a deeper understanding of how to optimize futsal training. Further studies could determine the best combination of these factors to maximize creativity in futsal players. The results of this study can be applied in training and sports environments to enhance players’ creativity and assist coaches in designing more innovative training programs.

Additionally, the environmental changes should be incorporated into training sessions regularly and with careful planning, ensuring that players are constantly exposed to new challenges that promote creativity. These changes should be introduced in a way that players are consistently faced with spatial limitations and varying conditions, but adequate rest periods must also be considered to prevent fatigue or confusion. For instance, coaches could introduce environmental modifications every few sessions or once a week to allow players to adapt effectively. At the same time, it's important to note that there could be some long-term negative effects, such as reduced focus or difficulty adjusting to fixed conditions in competition, so coaches must maintain a balance between environmental manipulation and traditional training methods. Furthermore, future research could explore the optimal combination of environmental changes, player numbers, and session durations to maximize creativity development.

## Data Availability

The raw data supporting the conclusions of this article will be made available by the authors, without undue reservation.

## Footnotes

^1^For this current study, we adhere to the original classification of Newell. From this perspective, this study will focus on task manipulation by changing the dimensions of the field (which implies changing rules), as well as on environmental manipulation by playing in futsal (i.e., indoor, solid surface, artificial light, …) versus soccer (outdoor, grass surface, sunlight,…) environment.

## Appendix

**Appendix 1:** Description of individual actions inferred from the study of Mohammadi Orangi et al. (8, 9). This is an exact copy of their study and we obtained permission to copy it earlier.

Category	Action	Description	
Pass (14)	With the inside dominant foot	Player passes with the inside of his dominant foot	
	With the outside dominant foot	Player passes with the outside of his dominant foot	
	Volley or with up-foot (dominant foot)	Player passes with up-foot of his dominant leg	
	Chipping pass with the dominant foot	Player passes with the toe of his dominant leg, so that, the ball rises from the ground	
	Heel pass with the dominant foot	Player passes with the heel of his leg	
	With the inside non-dominant foot	Player passes with the inside of his non-dominant foot	
	With the outside non-dominant foot	Player passes with the outside of his non-dominant foot	
	Volley or with up-foot (non-dominant)	Player passes with up-foot of his non-dominant leg	
	Chipping pass with non-dominant foot	Player passes with the toe of his non-dominant leg, so that, the ball rises from the ground	
	Heel pass with non-dominant foot	Player passes with the heel of his leg	
	Chest pass	Player passes with his chest	
	Head pass	Player passes with his head	
	Knee pass	Player passes with his knee	
	With other port of the body	Player passes with other parts of his body	
Dribble (9)	In the direction of the dominant foot	Player deceives the opponent by moving the ball in the direction of the inside dominant leg	
	In the indirection of the dominant foot	Player deceives the opponent by moving the ball in the indirection of inside dominant leg (outside foot)	
	Bilateral dribble	Player moves the ball from one side of the opponent and looking for the ball from the other side of him	
	Zidane dribble	Player stops the ball with the sole and goes around an opponent player using the other sole	
	Up-head dribble	Player passes the ball over the opponent's head and looks for the ball in that direction	
	Ronaldo dribble with the dominant foot	Player moves the ball with the inside of the dominant heel that is on the back of the non-dominant foot	
	Ronaldo dribble with non-dominant foot	Player moves the ball with the inside of the non-dominant heel that is on the back of the dominant foot	
	Changing direction	Player stands and moves in the other direction as he moves with high speed	
	With head	The player moves the ball to the other side of the opponent and follows it	
Shot (7)	With inside foot	Banana shot	The ball moves to one side then redirect. The ball goes in a semicircular way
		Non-banana	The ball goes in a straight direction
	With outside foot	Banana	The ball moves to one side then redirect. The ball goes in a semicircular way
		Non-banana	The ball goes in a straight direction
	With up -foot	The player kicks to the ball with his up-foot	
	Volley shot	Player's foot meets and directs the ball in an angled direction before it has time to reach the ground. In this case, the inner part of the foot is downward, and the angle of the foot that kicks is about 90° with the ground	
	Bicycle shot	Player kicks an airborne ball rearward in midair. It is achieved by throwing the body backward up into the air and, before descending to the ground, making a shearing movement with the lower limbs to get the ball-striking leg in front of the other	
kicking (12)	With the inside dominant foot	Player traps the ball with the inside of the dominant foot	
	With the outside dominant foot	Player controls the ball with the outside of the dominant foot	
	With the inside non-dominant foot	Player controls the ball with the inside of the non-dominant foot	
	With the outside non-dominant foot	Player controls the ball with the outside of the non-dominant foot	
	Up-foot reception (dominant)	Player controls the ball with the up-foot of the dominant leg	
	Up-foot reception (non-dominant)	Player controls the ball with the up-foot of a non-dominant leg	
	Marcelo stop	The player controls the ball (that's coming towards him) under his feet	
	Neymar stop (dominant foot)	The player controls the ball with the inside of the dominant heel that is on the back of the non-dominant foot	
	Neymar stop (non-dominant foot)	The player controls the ball with the inside of the non-dominant heel that is on the back of the dominant foot	
	With knee	Player controls the ball with his knee	
	With chest	Player controls the ball with his chest	
	With other parts of the body	Player controls the ball with other parts of his body	

**Appendix 2:** Overview of actions performed in different groups.

Variables	G1 pretest-post test (progress)	G2 pretest-post test (progress)	G3 pretest-post test (progress)	G4 pretest-post test (progress)
Total number (number)	608–748 (%23)	651–875 (%34)	594–813 (%36)	651–901 (%38)
Adequate (number)	257–337 (%31)	231–410 (%77)	254–385 (%51)	263–419 (%59)
Degree of variability (number)	92–133 (%44)	94–149 (%58)	108–131 (%21)	100–153 (%53)
Original (number)	4–13 (%225)	7–19 (%171)	7–12 (%71)	4–22 (%450)
Creative (number)	0–3 (%300)	2–11 (%450)	1–4 (%300)	1–14 (%1300)

The percentages in this table indicate the progress from pre-test to post-test.
